# Evaluation of healthcare professionals’ knowledge of Alzheimer’s disease in government hospitals and healthcare centers: A cross-sectional study in Jeddah, Saudi Arabia

**DOI:** 10.1097/MD.0000000000041642

**Published:** 2025-02-28

**Authors:** Yahya A. Alzahrani, Maan H. Harbi, Adel G. Almalki, Rana A. Althomali, Roaa F. Alorabi, Bashaer S. Basulyman, Khawla A. Alansari, Jasser A. Alzahrani, Dalia A. Alzahrani, Ibrahim A. Asuhaimi, Abdullah M. Alzahrani, Ayed A. Alkatheeri

**Affiliations:** a Department of Pharmacy, East Jeddah Hospital, Ministry of Health, Jeddah, Saudi Arabia; b King Abdullah International Medical Research Center, Jeddah, Saudi Arabia; c Pharmacology and Toxicology Department, College of Pharmacy, Umm Al-Qura University, Makkah, Saudi Arabia; d Pharmaceutical Care Department, Ministry of National Guard – Health Affairs, Jeddah, Saudi Arabia.

**Keywords:** Alzheimer disease, attitudes, health personnel, knowledge, practice, Saudi Arabia

## Abstract

Knowledge of Alzheimer’s disease (AD) among healthcare staff influences important aspects of care and is associated with better patient outcomes. This study aimed to assess the knowledge of AD among healthcare professionals working in government hospitals and healthcare centers in Jeddah, Saudi Arabia. This cross-sectional study used an online questionnaire to assess Alzheimer’s disease knowledge using the Alzheimer’s Disease Knowledge Scale (ADKS). Participants also self-rated their knowledge and reported their experience in dementia care and training. The study included 231 healthcare professionals, with 61.5% males and 38.5% females. Medical professionals demonstrated significantly higher mean ADKS score = 19.84 compared to pharmacy, nursing, and allied health professionals (*P* < .05). Other factors, such as gender, age, workplace setting, years of experience, and formal dementia training, showed no significant impact on ADKS scores (*P* > .05). However, participants with additional dementia-related learning experiences, such as workshops or self-directed study, had significantly higher scores (*P* < .05). A notable finding was the discrepancy between self-assessed and actual knowledge. Among those who rated themselves as “very knowledgeable,” only 10% achieved a very good ADKS score, while more than half failed the test. Conversely, those who rated themselves as having poor knowledge often performed better than expected, with many achieving good or very good ADKS scores 44.3% and 13.1%, respectively. This study highlights significant disparities in Alzheimer’s disease knowledge among healthcare professionals in Jeddah, with medical staff showing higher understanding. Tailored educational programs are needed to address these gaps and improve dementia care.

## 1. Introduction

Alzheimer’s disease is a progressive brain disorder that leads to gradual cognitive decline, memory loss, and the inability to perform everyday tasks. It is the most common form of dementia among elderly populations worldwide. Several theories have been proposed regarding the causes of Alzheimer’s disease (AD), including the amyloid cascade hypothesis and the tau hypothesis.^[1,2]^

The incidence of AD continues to rise globally, due not only to the aging population but also to changing lifestyles.^[3,4]^ In Saudi Arabia, the prevalence of dementia is estimated to be approximately 5%, with a noticeable increase in people over age 65.^[4,5]^ The economic burden of dementia is substantial, with global costs estimated at more than $1.3 trillion and expected to rise to $2 trillion by 2030.^[6]^

When individuals are affected by AD, their families and caregivers face additional responsibilities and duties, such as seeking and dealing with healthcare providers, negotiating medications, monitoring patient behavior, and attending to the patient’s daily needs.^[7,8]^ However, the literature suggests that healthcare practitioners are not well trained regarding the early signs, course, and management of AD.^[9]^ There is a significant lack of knowledge among general practitioners and healthcare workers, especially those who are not geriatric specialists, which negatively affects the quality of care.^[9]^

In the acute care setting, where 15% to 50% of patients have some degree of cognitive impairment, knowledge of Alzheimer’s among those involved in their care is essential.^[10]^ Even staff members who have minimal contact with patients (e.g., cleaning, maintenance, and security personnel) would benefit from greater general knowledge about dementia. Acute care nurses without an accurate understanding of AD have admitted to having difficulty interpreting behaviors, prioritizing care for those without Alzheimer’s, and experiencing fear of caring for patients with Alzheimer’s due to perceived risks.^[11]^ Previous research has shown that providing high-quality care to patients with Alzheimer’s in an acute care setting is achievable when their specific needs are appropriately addressed.^[12]^ Two studies were conducted to corroborate these points about the relationship between knowledge of Alzheimer’s and quality of care in the acute care environment. Both sets of authors concluded that staff education and training were critical to improving the quality of care received by a person with Alzheimer’s while in acute care.^[13,14]^

Knowledge of AD among healthcare staff influences important aspects of care, such as the timing of diagnosis and the implementation of interventions, which are associated with better patient outcomes.^[7,15–20]^ Assessment of healthcare staff’s present level of knowledge about Alzheimer’s using a reliable tool is the first step toward improving Alzheimer’s knowledge for all types of healthcare staff.

Over the last decade, several tools have been developed to assess Alzheimer’s knowledge, including the Alzheimer’s Disease Knowledge Scale (ADKS), which has demonstrated strong reliability and validity in measuring knowledge about the disease.^[9]^ However, no study has been conducted to assess the knowledge of AD among healthcare staff in government hospitals in Jeddah, Saudi Arabia. This lack of data highlights the need for targeted educational programs to improve the quality of care provided to Alzheimer’s patients. This study thus aims to identify the level of awareness regarding AD in the healthcare staff working across governmental hospitals and healthcare centers in Jeddah, Saudi Arabia, using the ADKS.

## 2. Materials and methods

### 2.1. Study design

This cross-sectional study, conducted between March 2023 and August 2023, included all healthcare staff actively working in Ministry of Health government hospitals and primary healthcare centers in Jeddah, Saudi Arabia.

Questionnaire (Appendix 1, Supplemental Digital Content, http://links.lww.com/MD/O429):

1. Demographic and background questions: The survey included demographic questions on topics such as gender, age group, family history of dementia, professional group (physicians, dentists, pharmacists, nurses, and allied health professionals), work setting (hospital or primary healthcare centers), and years of experience. Moreover, 2 additional questions gauged participants’ experience in caring for people with dementia: 1 focused on non-work-related personal caregiving and the other on professional caregiving.

To measure dementia-specific training or education, participants selected from 7 options, grouped into 3 categories:

Tertiary education: those who had completed undergraduate or postgraduate courses covering Alzheimer’s content.
Alzheimer’s training: those who had attended a dementia-specific conference, a hospital in-service course on Alzheimer’s, or a workshop or session run by the Saudi Alzheimer’s Disease Association.
Other Alzheimer’s-related learning: those who reported self-directed learning (e.g., online content or reading) or other informal learning experiences related to dementia.

2. Alzheimer’s Disease Knowledge Scale (ADKS): The ADKS was used to assess AD knowledge due to its ease of use, reliability, and validity. It consists of 30 true/false items, and the final score reflects the number of correct responses. The ADKS had a test-retest reliability correlation of 0.81 and an internal consistency score (Cronbach’s α) of 0.71. Content and predictive validity were deemed adequate. The ADKS is conceptually divided into 7 content domains: life impact (items 1, 11, 28), risk factors (items 2, 13, 18, 25, 26, 27), symptoms (items 19, 22, 23, 30), treatment and management (items 9, 12, 24, 29), assessment and diagnosis (items 4, 10, 20, 21), caregiving (items 5, 6, 7, 15, 16), and course of the disease (items 3, 8, 14, 17). The scale took approximately 5 to 10 minutes to complete.
3. Self-reported Alzheimer’s knowledge: Participants were asked to rate their own level of Alzheimer’s knowledge using 5 categories: very knowledgeable, good, average, poor, and no knowledge. This self-assessment was later compared to their scores on the ADKS, which were classified into 4 grade categories: very good, good, pass, and fail.

### 2.2. Sample size calculation

The sample size was determined using a single population proportion formula:


$$\begin{array}{l} n=Z{\left(\frac{\alpha }{2}\right)}^{2}\times \frac{P\left(1-P\right)}{D^{2}} \end{array}$$

Whereas n = sample size; *Z* = (α/2) = 1.96; *P* = .5; *D* = marginal error (5%). n = 384 patients.

### 2.3. Process and data collection

The survey was distributed online using SurveyMonkey software. A mass email containing the survey link was sent to all staff in government hospitals and primary healthcare centers in Jeddah, Saudi Arabia via their internal email systems. To maximize the response rate, an initial email describing the study was sent, followed by 2 reminder emails, all of which included the survey link. The survey was accessible for approximately 4 weeks, after which data were collected.

### 2.4. Ethical approval

The study received ethical approval from the Medical Research and Studies Department of the Directorate of Health Affairs, Jeddah, Saudi Arabia, under approval number A00634.

### 2.5. Endpoints

#### 2.5.1. Primary endpoints

- To assess the overall ADKS scores across healthcare professional backgrounds, including medicine, nursing, pharmacy, and allied health.

#### 2.5.2. Secondary endpoints

- To evaluate differences in ADKS domain-specific scores (e.g., life impact, assessment, and caregiving) across various professional backgrounds.
- To examine the correlation between self-rated knowledge levels and actual ADKS performance to identify gaps in self-awareness among healthcare professionals.

### 2.6. Statistical analysis

Data were analyzed using the Statistical Package for Social Science (SPSS) program, version 23. Descriptive statistics, such as frequency, percentage, mean, and standard deviation (SD), were used to analyze demographic data, and weighted means were calculated for self-reported knowledge. ADKS scores across groups were compared using independent *t* tests and ANOVA tests. All reported *P* values were 2-sided; a *P* value of <.05 was considered statistically significant.

## 3. Results

### 3.1. Influence of background characteristics on dementia knowledge among healthcare professionals

The study included 231 healthcare professionals, comprising 142 males (61.5%) and 89 females (38.5%). Participants were categorized into 3 age groups: under 30 years (19.9%), 30 to 50 years (70.6%), and over 50 years (9.5%). The professional backgrounds of the participants were diverse, with 19.5% working in medicine, 30.3% in pharmacy, 30.3% in nursing, and 19.9% in allied health. The majority of participants (89.2%) were employed in hospital settings, while 10.8% worked in primary health facilities. Regarding their professional experience, participants had varied years of experience, ranging from <1 year to over 20 years. Additionally, 17.3% had a family history of dementia, and 18.2% had personal caring experience with dementia patients. Professional caring experience with dementia patients was reported by 19% of participants. Only 8.2% had tertiary education, while 10.4% had received dementia training, and 61.9% had other forms of dementia learning.

Analysis revealed no significant difference in dementia knowledge between males and females, with males achieving a mean ADKS score of 17.94 (SD = 2.59) and females slightly higher at 18.18 (SD = 3.70), indicated by a *P* value of 0.570. Age also did not show a significant effect on dementia knowledge (*P* = .230), although participants over 50 years had the highest mean score of 19.09 (SD = 2.56), compared to 17.82 (SD = 2.89) for those under 30 years and 17.95 (SD = 3.16) for those aged 30 to 50 years. Interestingly, the professional group demonstrated a significant relationship with dementia knowledge (*P* < .05), with medical professionals scoring the highest (mean = 19.84, SD = 2.98), followed by pharmacy, nursing, and allied health professionals. Conversely, workplace settings (hospital vs primary health facility) did not show a significant difference (*P* = .485). Years of experience, whether participants had a family history of dementia, personal or professional caring experience with dementia patients, and tertiary education also showed no significant impact on dementia knowledge. Furthermore, dementia training did not significantly affect knowledge scores (*P* = .256). However, other forms of dementia learning were significantly associated with higher dementia knowledge (*P* < .05), with those having additional learning experiences about dementia achieving a mean score of 18.46 (SD = 3.11), compared to 17.34 (SD = 2.87) for those without such learning (Table 1).

**Table 1:** Background characteristics and their relationship to dementia knowledge (N = 231).

Background characteristics	n (%)	ADKS mean (SD)	Significance
Demographics			
Gender (n = 231)			*P* = .570
Male	142 (61.5 %)	17.94 (2.59)	
Female	89 (38.5%)	18.18 (3.70)	
Age			*P* = .230
<30 yr	46 (19.9 %)	17.82 (2.89)	
30–50 yr	163 (70.6 %)	17.95 (3.16)	
<50 yr	22 (9.5%)	19.09 (2.56)	
Professional group			*P*<.05
Medicine	45 (19.5%)	19.84 (2.98)	
Pharmacy	70 (30.3%)	17.58 (2.98)	
Nurse	70 (30.3%)	17.95 (3.06)	
Allied health	46 (19.9%)	17.07 (2.61)	
Workplace			*P* = .485
Hospital	206 (89.20%)	2.61 (3.12)	
Primary health facility	25 (10.80%)	18.44 (2.38)	
Experience and education			
Years of experience			*P* = .499
>1 yr	7 (3%)	18.57 (3.59)	
1–5 yr	53 (22.9%)	17.58 (2.87)	
5–10 yr	58 (25.1%)	17.77 (3.30)	
10–15 yr	59 (25.5%)	18.07 (2.59)	
15–20 yr	27 (11.7%)	18.93 (3.88)	
>20 yr	27 (11.7%)	18.37 (2.88)	
Family history of dementia?			*P* = .409
Yes	40 (17.3%)	18.82 (3.12)	
No	191 (82.7%)	17.87 (3.04)	
Personal caring experience with the dementia patient			*P* = .073
Yes	42 (18.2%)	18.31 (2.67)	
No	189 (81.8%)	17.97 (3.15)	
Professional caring experience with the dementia patient			*P* = .522
Yes	44 (19%)	18.31 (3.24)	
No	187 (81%)	18.03 (3.06)	
Tertiary education			*P* = .978
Yes	19 (8.2%)	18.05 (2.75)	
No	212 (91.8%)	17.87 (3.11)	
Dementia training			*P* = .256
Yes	24 (10.4%)	18.70 (2.88)	
No	207 (89.6%)	17.96 (3.08)	
Other dementia learning			*P* < .05
Yes	143 (61.9%)	18.46 (3.11)	
No	88 (38.1%)	17.34 (2.87)	

Values are presented as frequency (percentage) and mean ± standard deviation (SD). *P* < .05 denotes statistical significance by independent *t* test and 1-way ANOVA.

ADKS: Alzheimer’s Disease Knowledge Scale.

### 3.2. Domain-specific analysis of Alzheimer’s disease knowledge among healthcare professionals

The study further investigated AD knowledge across various domains for healthcare professionals from various disciplines, including medicine, nursing, pharmacy, and allied health. The ADKS scores were evaluated for life impact, risk factors, symptoms, treatment management, assessment, caregiving, and the course of the disease.

In the domain of life impact, significant differences were observed (*P* < .05), with the medicine group scoring higher (mean = 2.51, SD = 0.63) compared to allied health (mean = 2.09, SD = 0.81) and nursing (mean = 2.05, SD = 0.75). Additionally, in the assessment domain, significant differences were noted (*P* < .05), with the medicine group scoring the highest (mean = 3.29, SD = 0.82) compared to the pharmacy group (mean = 2.73, SD = 0.88). Moreover, in the caregiving domain, significant differences were also observed (*P* < .05), with the medicine group having the highest mean score (mean = 2.80, SD = 1.06) compared to the nursing group (mean = 2.31, SD = 0.91). In the risk factors, symptoms, treatment, and course of disease domains, no significant differences were found (*P* = .11, 0.05, 0.20, and 0.11, respectively; Table 2).

**Table 2:** ADKS content domains and professional group.

Domain	Medicine	Pharmacy	Nursing	Allied health	*P* value
	Mean ± SD	Mean ± SD	Mean ± SD	Mean ± SD	
Life impact	2.51 ± 0.63	2.21 ± 0.74	2.05 ± 0.75*	2.09 ± 0.81*	<.05
Risk factors	2.84 ± 1.17	2.67 ± 1.28	3.04 ± 1.19	2.52 ± 1.15	.11
Symptoms	2.91 ± 0.79	2.52 ± 0.85	2.50 ± 0.93	2.47 ± 0.86	<.05
Treatment management	2.47 ± 0.79	2.30 ± 0.73	2.37 ± 0.89	2.11 ± 0.92	.20
Assessment	3.29 ± 0.82	2.73 ± 0.88*	2.90 ± 0.95	2.83 ± 0.93	<.05
Caregiving	2.80 ± 1.06	2.59 ± 0.92	2.31 ± 0.91*	2.33 ± 0.92	<.05
Course of disease	3.02 ± 1.03	2.56 ± 1.03	2.77 ± 0.89	2.72 ± 1.03	.11

Values are presented as mean ± standard deviation (SD).

ADKS = Alzheimer’s Disease Knowledge Scale, SD = standard deviation.

* *P* < .05 denotes statistical significance compared with the corresponding medicine group by 1-way ANOVA.

### 3.3. Distribution of ADKS scores based on self-rated knowledge levels

**Figure 1.**
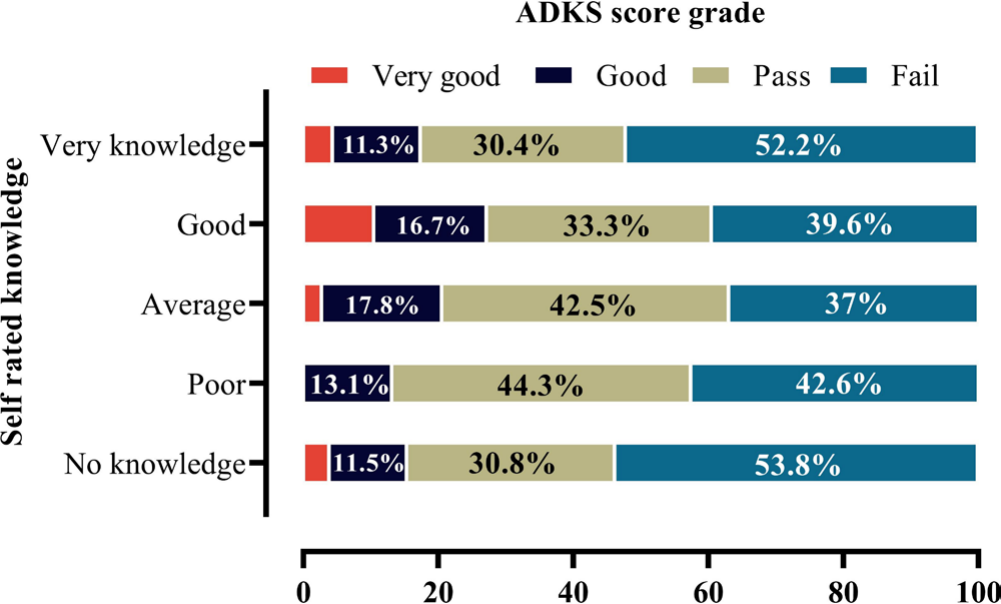
Distribution of Alzheimer’s Disease Knowledge Scale (ADKS) Scores Across Self-Rated Knowledge Levels. ADKS = Alzheimer’s Disease Knowledge Scale.

Figure 1 illustrates the distribution of ADKS scores across various levels of self-rated knowledge. It details the percentage of individuals achieving each ADKS score (i.e., very good, good, pass, fail) within each category of self-assessed knowledge, namely very knowledgeable, good, average, poor, and no knowledge.

Among participants who rated themselves as very knowledgeable, <10% achieved a very good grade, 11.3% attained a good grade, 30.4% obtained a passing grade, and the majority (52.2%) failed. For those with a self-assessed good knowledge level, 16.7% earned a very good grade, 33.3% secured a good grade, 39.6% passed, and 10.4% failed. In the average knowledge category, 17.8% received a very good grade, 42.5% achieved a good grade, 37% obtained a pass grade, and 2.7% failed. Participants who rated their knowledge as poor demonstrated that 13.1% achieved a very good grade, 44.3% obtained a good grade, and 42.6% passed; there were no failures. Lastly, among individuals who claimed no knowledge, 11.5% attained a very good grade, 30.8% achieved a good grade, 53.8% passed, and 3.9% failed (Table 2).

## 4. Discussion

This study has identified several important features about the perceptions of dementia and the factors that determine the level of knowledge in each type of health professional. These findings align with the study’s objective to evaluate disparities in Alzheimer’s knowledge among healthcare professionals, emphasizing the need for targeted and improved educational interventions in order to prepare healthcare workers for the dementia challenge.

A salient finding was the variation that existed in dementia knowledge across professions. Medical professionals scored the highest knowledge, statistically higher than the pharmacy, nursing, and allied health scores. This finding supports that the medical profession receives greater focused training about dementia than others, particularly those from the nursing and allied health professions, who may receive less extensive education.^[9,10]^ With dementia becoming more prevalent, it is crucial that healthcare workers of all professional backgrounds possess appropriate knowledge regarding the illness. Tailor-made training programs regarding the needs of non-medical professionals may help to close this gap and improve the care of patients with dementia.

The observed differences in knowledge levels across professions underscore the necessity for tailored educational interventions. Studies have shown that healthcare workers lacking adequate training in dementia care can negatively impact patient outcomes.^[7,11]^ A pressing need exists for comprehensive training programs aimed at enhancing dementia knowledge among nursing and allied health professionals. Conversely, other literature suggests that interdisciplinary training could bridge knowledge gaps,^[13,14]^ advocating for collaborative learning environments to enhance overall care quality.

Surprisingly, formal dementia training did not significantly enhance participants’ knowledge. A wide range of other learning experiences, such as attending workshops, case analyses, and self-initiated learning, are associated with higher scores. This finding may indicate that conventional formal training methods are not as successful as we think. This finding contradicts previous studies that advocate for structured educational programs as effective means to improve knowledge.^[12]^ The association of higher scores with diverse learning experiences (e.g., workshops and self-directed learning) suggests that traditional training methods may need reevaluation. Innovative and flexible learning opportunities could enhance engagement and retention of critical information among healthcare workers.^[17]^ Innovative training can include short app-based modules and interactive e-learning sessions that fit into daily routines, allowing for continuous learning without disrupting workflows. Monthly workshops or tele-mentoring sessions can complement these by offering hands-on experience, case-based discussions, and real-time feedback from experts. Partnering with professional organizations and offering incentives like continuing education credits can further encourage participation, ensuring that training remains practical, engaging, and impactful in improving patient care. Future research could investigate new methods impact on the improvement of knowledge of care providers.

Considering professions, it is clear that there are several key differences in areas of dementia knowledge. Scores for the 3 components comprising the knowledge model were higher for medical professionals: understanding the life impact of dementia, assessing patients, and caregiving support. Because these are key dimensions of dementia care, it is of concern that other professional groups’ scores are so much lower for these components, which further highlights the need for more focused education in these areas, particularly among nursing and allied health professionals. The focus on these areas of deficits will ensure that all healthcare providers are suitably prepared to provide comprehensive and sensitive care to dementia patients. This finding is consistent with previous research indicating that healthcare providers often lack essential knowledge required for effective dementia care.^[8,20]^ Addressing these gaps through focused education is imperative for improving care quality.

A striking finding from this study is the tendency among healthcare staff to overestimate their knowledge of AD. Despite self-reported high levels of confidence, the actual scores on the ADKS reveal a disconnect between perceived and actual knowledge. This phenomenon is not unique to this study; it has been documented in healthcare settings globally, suggesting a widespread issue across different professional groups. In regard to self-assessment versus actual knowledge, the results indicate that a significant proportion of participants who rated themselves as “very knowledgeable” did not perform well on the ADKS. For instance, <10% of those who considered themselves very knowledgeable achieved a “very good” grade on the ADKS, while over half failed. This discrepancy highlights a critical gap in self-awareness regarding dementia knowledge among healthcare professionals. Similar findings have been reported in other studies, where healthcare workers often overestimate their understanding of complex medical conditions, leading to inadequate patient care.^[21,22]^

### 4.1. Implications for patient care

The overestimation of knowledge may have serious implications for patient care. When healthcare professionals believe they possess sufficient knowledge about AD, they may not seek further education or training that is essential for effective patient management. This finding aligns with previous research indicating that healthcare providers with inflated self-assessments are less likely to engage in continuing education.^[23]^ Consequently, this situation can lead to suboptimal care practices, poor communication with patients and families, and, ultimately, worse health outcomes for individuals living with dementia.^[7,19,20]^

To address this issue, there is an urgent need for improved educational strategies that not only enhance knowledge but also foster accurate self-assessment among healthcare professionals. Incorporating reflective practices and feedback mechanisms into training programs could help individuals better understand their level of knowledge and identify areas needing improvement.^[14]^ Furthermore, utilizing assessment tools such as the ADKS before and after educational interventions could provide valuable insights into the effectiveness of training programs and ensure that staff are adequately prepared to meet the needs of patients with AD.

### 4.2. Limitations and future research

This study has certain limitations, particularly regarding participation rates, which may affect the generalizability of the findings. While the target sample size was 384 participants, data were collected from only 231 respondents, resulting in a response rate of approximately 60%. This reduced sample size may have limited the statistical power to detect significant differences, especially in subgroup analyses, and increased the risk of type II errors, where true associations could have been missed.

To improve participation in future research, strategies such as leveraging digital platforms (e.g., mobile apps, social media, and online surveys) could enhance accessibility and engagement. Additionally, flexible participation options, including asynchronous data collection and hybrid study designs, may help reach a broader and more diverse sample. Despite the smaller sample, the findings provide valuable preliminary insights. Future studies should prioritize enhanced recruitment strategies to achieve the intended sample size, improve representativeness, and ensure adequate statistical power.

Given the multiple statistical comparisons conducted, the possibility of type I errors should be considered, and results interpreted with caution. Replication in larger, more diverse samples is necessary to confirm these findings.

In addition, it is a cross-sectional study; hence, it reflects a snapshot in time about dementia knowledge and cannot provide an indication of how that knowledge changes over time. Additionally, the self-reported nature of the survey may have introduced response bias, potentially overestimating self-assessed knowledge. Additionally, the reliance on voluntary participation might limit the representativeness of the sample, introducing selection bias. In this field, research into knowledge gaps should be expanded to larger and more diverse populations and possibly with longitudinal designs that allow the tracking of changes in dementia knowledge over time with continued training and experience.

## 5. Conclusion

This study highlights critical insights into the knowledge of AD among healthcare staff in Jeddah, Saudi Arabia, revealing significant disparities across professional groups. Medical professionals demonstrated a higher level of understanding compared to nursing, pharmacy, and allied health staff, emphasizing the need for tailored educational interventions to address specific knowledge gaps. However, formal dementia training did not significantly enhance knowledge levels; instead, diverse learning experiences such as workshops and self-directed learning were associated with better outcomes. This finding suggests that traditional training methods may require reevaluation in favor of more innovative and flexible educational approaches that encourage deeper engagement.

Additionally, the study identified specific areas where medical professionals excelled, including understanding the life impact of dementia, patient assessment, and caregiving support. These dimensions are crucial for providing quality care and highlight the urgent need for focused education among nursing and allied health professionals. By addressing these knowledge deficits through targeted training programs, healthcare systems can better equip all professionals to meet the challenges posed by AD, ultimately improving patient outcomes and care quality for individuals living with dementia.

## Acknowledgments

The authors extend their appreciation to Umm Al-Qura University, Saudi Arabia for funding this research work through grant number: 25UQU4310027GSSR01.

## Author contributions

**Conceptualization:** Yahya A. Alzahrani, Jasser A. Alzahrani.

**Data curation:** Adel G. Almalki, Bashaer S. Basulyman, Khawla A. Alansari, Jasser A. Alzahrani, Dalia A. Alzahrani, Ibrahim A. Asuhaimi.

**Formal analysis:** Dalia A. Alzahrani, Ibrahim A. Asuhaimi.

**Writing – original draft:** Maan H. Harbi, Abdullah M. Alzahrani, Ayed A. Alkatheeri.

**Writing – review & editing:** Maan H. Harbi, Rana A. Althomali, Roaa F. Alorabi, Ayed A. Alkatheeri.

## Supplementary Material


