# Supplementary material for: Evaluation of healthcare professionals’ knowledge of Alzheimer’s disease in government hospitals and healthcare centers: A cross-sectional study in Jeddah, Saudi Arabia
Source: Medicine (Baltimore). 2025 Feb 28;104(9):e41642. doi: 10.1097/MD.0000000000041642 (PMC11875628; doi:10.1097/MD.0000000000041642)
Supplement: Supplementary file 1 [file medi-104-e41642-s001.docx]

**Appendix 1: Survey Questionnaire**

| **1- Demographic and background questions:** | | | | | | | | | | | | | | |
| --- | --- | --- | --- | --- | --- | --- | --- | --- | --- | --- | --- | --- | --- | --- |
| **Gender** | | **🞅 Male** | | | | | | | | | | **🞅 Female** | | |
| Age | **🗌 <30 years** | | | **30-50 years** | | | | | **>50 years** | | | | | |
| Professional Group | Medicine | | Pharmacy | | Nursing | | | Allied health service (Psychologist, Occupational therapist, Dietitian, Speech pathologist, Physiotherapist , Audiologist and social worker) | | | | | | |
| Workplace | Hospital | | | | | | primary health care center | | | | | | | |
| Years of experience | 🗌Less than 1 year 🗌1–5 years 🗌5–10 years  🗌10–15 years 🗌15–20 years 🗌More than 20 years | | | | | | | | | | | | | |
| **2-Self-reported dementia knowledge:** | | | | | | | | | | | | | | |
| Do you have a family history of dementia? | | | | | | | | | | | Yes | | | No |
| Do you have a personal caring experience with dementia's patient? | | | | | | | | | | | Yes | | | No |
| Do you have a professional caring experience with dementia's patient? | | | | | | | | | | | Yes | | | No |
| Have you taken an undergraduate or postgraduate course on dementia? | | | | | | | | | | | Yes | | | No |
| Have you attended a dementia-specific conference, a hospital in-service Alzheimer course, or a workshop run by Saudi Alzheimer's disease association? | | | | | | | | | | | Yes | | | No |
| Have you read online content about dementia? | | | | | | | | | | | Yes | | | No |
| On a scale of 0 to 10, how high would you rate your knowledge about dementia? | | | | | | Very knowledgeable  good  Average  Poor  No knowledge. | | | | | | | | |
| **3-Alzheimer’s disease knowledge scale** | | | | | | | | | | | | | | |
| 1. People with Alzheimer’s disease are particularly prone to depression. | | | | | | | | | | True | | | False | |
| 1. It has been scientifically proven that mental exercise can prevent a person from getting Alzheimer’s disease. | | | | | | | | | | True | | | False | |
| 1. After symptoms of Alzheimer’s disease appear, the average life expectancy is 6 to 12 years. | | | | | | | | | | True | | | False | |
| 1. When a person with Alzheimer’s disease becomes agitated, a medical examination might reveal other health problems that caused the agitation. | | | | | | | | | | True | | | False | |
| 1. People with Alzheimer’s disease do best with simple, instructions given one step at a time. | | | | | | | | | | True | | | False | |
| 1. When people with Alzheimer’s disease begin to have difficulty taking care of themselves, caregivers should take over right away. | | | | | | | | | | True | | | False | |
| 1. If a person with Alzheimer’s disease becomes alert and agitated at night, a good strategy is to try to make sure that the person gets plenty of physical activity during the day. | | | | | | | | | | True | | | False | |
| 1. In rare cases, people have recovered from Alzheimer’s disease. | | | | | | | | | | True | | | False | |
| 1. People whose Alzheimer’s disease is not yet severe can benefit from psychotherapy for depression and anxiety. | | | | | | | | | | True | | | False | |
| 1. If trouble with memory and confused thinking appears suddenly, it is likely due to Alzheimer’s disease. | | | | | | | | | | True | | | False | |
| 1. Most people with Alzheimer’s disease live in nursing homes. | | | | | | | | | | True | | | False | |
| 1. Poor nutrition can make the symptoms of Alzheimer’s disease worse | | | | | | | | | | True | | | False | |
| 1. People in their 30s can have Alzheimer’s disease. | | | | | | | | | | True | | | False | |
| 1. A person with Alzheimer’s disease becomes increasingly likely to fall down as the disease gets worse. | | | | | | | | | | True | | | False | |
| 1. When people with Alzheimer’s disease repeat the same question or story several times, it is helpful to remind them that they are repeating themselves. | | | | | | | | | | True | | | False | |
| 1. Once people have Alzheimer’s disease, they are no longer capable of making informed decisions about their own care. | | | | | | | | | | True | | | False | |
| 1. Eventually, a person with Alzheimer’s disease will need 24-hour supervision. | | | | | | | | | | True | | | False | |
| 1. Having high cholesterol may increase a person’s risk of developing Alzheimer’s disease. | | | | | | | | | | True | | | False | |
| 1. Tremor or shaking of the hands or arms is a common symptom in people with Alzheimer’s disease | | | | | | | | | | True | | | False | |
| 1. Symptoms of severe depression can be mistaken for symptoms of Alzheimer’s disease. | | | | | | | | | | True | | | False | |
| 1. Alzheimer’s disease is one type of dementia. | | | | | | | | | | True | | | False | |
| 1. Trouble handling money or paying bills is a common early symptom of Alzheimer’s disease. | | | | | | | | | | True | | | False | |
| 1. One symptom that can occur with Alzheimer’s disease is believing that other people are stealing one’s things. | | | | | | | | | | True | | | False | |
| 1. When a person has Alzheimer’s disease, using reminder notes is a crutch that can contribute to decline. | | | | | | | | | | True | | | False | |
| 1. Prescription drugs that prevent Alzheimer’s disease are available. | | | | | | | | | | True | | | False | |
| 1. Having high blood pressure may increase a person’s risk of developing Alzheimer’s disease. | | | | | | | | | | True | | | False | |
| 1. Genes can only partially account for the development of Alzheimer’s disease. | | | | | | | | | | True | | | False | |
| 1. It is safe for people with Alzheimer’s disease to drive, as long as they have a companion in the car at all times. | | | | | | | | | | True | | | False | |
| 1. Alzheimer’s disease cannot be cured. | | | | | | | | | | True | | | False | |
| 1. Most people with Alzheimer’s disease remember recent events better than things that happened in the past. | | | | | | | | | | True | | | False | |
